# Maternal Nutritional Status Governs Fetal Development by Modulating Imprinting Gene GAB1‐Mediated Trophoblast Differentiation in the Placenta

**DOI:** 10.1111/cpr.70069

**Published:** 2025-06-23

**Authors:** Mingming Fan, Hongyu Wu, Yuan Xie, Ming Liu, Xin Yu, Feiyang Wang, Zhenyu Xiao, Hongmei Wang, Xuan Shao, Yan‐Ling Wang

**Affiliations:** ^1^ State Key Laboratory of Organ Regeneration and Reconstruction Institute of Zoology, Chinese Academy of Sciences Beijing China; ^2^ University of Chinese Academy of Sciences Beijing China; ^3^ International Society of Zoological Sciences Beijing China; ^4^ Beijing Institute for Stem Cell and Regenerative Medicine Beijing China; ^5^ School of Life Science Beijing Institute of Technology Beijing China

**Keywords:** autophagy, fetal growth restriction, food restriction, GAB1, placental trophoblast, syncytialisation

## Abstract

The appropriate allocation of nutrients between the mother and the fetus during mammalian pregnancy primarily depends on a healthy placenta. Fetal growth restriction (FGR) is frequently associated with inadequate maternal nutrition supply and impaired placental function. The precise mechanisms linking maternal nutrient deficiency to compromised fetal and placental development remain largely elusive. In this study, we conducted an in‐depth analysis by integrating single‐cell/single‐nucleus RNA sequencing data from human and mouse placentas along with transcriptomic data from FGR placenta, identifying the *GAB1* (GRB2‐associated binding protein 1) gene as a potential mediator of dysregulated maternal–fetal exchange, thereby affecting fetal growth. Using a mouse model, we demonstrated that food restriction significantly impeded fetal growth and disrupted placental labyrinth development. Through an in vitro trophoblast differentiation model, we revealed that nutritional restriction impaired GAB1 stability via LC3‐interacting region (LIR) motif‐mediated selective autophagic degradation, thereby hindering GAB1‐MAPK signalling‐enhanced trophoblast syncytialisation. These findings elucidate the mechanisms by which placental GAB1 links maternal nutrition status with fetal growth and suggest potential therapeutic strategies for managing pregnancy complications such as FGR.

## Graphical abstract

Maternal nutrient restriction disrupts placental homeostasis via GAB1 dysregulation—involving both transcriptional downregulation and autophagy‐dependent protein degradation. These molecular alterations inhibit MAPK signalling‐mediated trophoblast fusion, leading to structural and functional deficits in the maternal–fetal exchange interface.

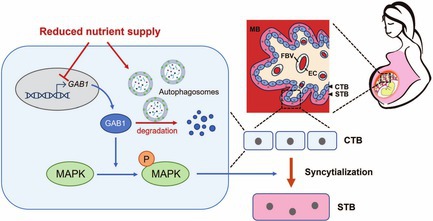

## 1 Introduction

Fetal growth restriction (FGR), defined as a pregnancy complication wherein the fetus fails to achieve its genetically determined growth potential [1, 2], is one of the leading causes of perinatal morbidity and mortality [3, 4]. Approximately 30 million newborns annually, particularly those in developing countries, are affected by FGR. The primary cause of FGR is often attributed to inadequate maternal nutrition, which compromises placental functions leading to an unfavourable intrauterine environment for fetal development [5, 6, 7]. Emerging evidence from the Developmental Origins of Health and Disease (DOHaD) framework suggests that maternal malnutrition or stress exposure during pregnancy significantly increases the risk of adult‐onset metabolic and cardiovascular disorders in offspring [8], and FGR represents a key manifestation of the DOHaD paradigm [3, 4]. However, the precise mechanisms linking maternal nutrient deficiency to impaired fetal and placental development remain largely elusive.

The appropriate allocation of nutrients between the mother and the fetus primarily depends on the placenta, which facilitates the transfer of materials between the two entities. Extensive research has highlighted the crucial role of the placenta in sensing fetal nutritional demands, modulating maternal supply and adapting its nutrient transport capacity [9, 10, 11]. The primary functional unit of the placenta is the villous tree structure, which in humans consists of an outer layer of multinucleated syncytiotrophoblasts (STBs) and an inner layer of mononucleated cytotrophoblast cells (CTBs) [12, 13, 14, 15]. STBs are formed through the fusion of CTBs, a process known as syncytialisation. This cell differentiation process is conserved in the mouse placenta, where labyrinth mononucleated trophoblast cells differentiate into two layers of STBs via cell–cell fusion [16]. The syncytial layer is directly bathed in maternal blood, positioning it to regulate feto‐maternal exchanges of gases, nutrients and waste products [12, 13, 14, 15]. Previous studies have shown that placentas from FGR foetuses exhibit impaired syncytialisation, particularly in cases of severe maternal nutritional deprivation [17, 18, 19]. Our recent research demonstrates that the mechanistic target of rapamycin (mTOR)‐TFEB signalling pathway in the placenta senses maternal nutritional status and modulates trophoblast syncytialisation and macropinocytotic activity, thereby compensating for the detrimental effects of maternal amino acid deficiency [1, 2]. Consequently, further investigations into the molecular mechanisms linking maternal nutritional stress to abnormal placental development are essential for understanding the pathological causes of FGR.

Importantly, conflict theory has elucidated a complex relationship characterised by both cooperation and competition between the mother and her fetus during pregnancy [12, 20]. The appropriate activities of imprinted genes are pivotal in mediating this maternal–fetal conflict by influencing placental development, metabolism and nutrient allocation [21, 22]. These genes are unique in that only one allele, inherited from either parent, undergoes epigenetic repression, leading to monoallelic expression [21, 22]. Studies using genetically manipulated mice have demonstrated that maternally expressed genes such as *H19, Igf2r* and *Phlda2* tend to restrict nutrient transfer to the fetus, while paternally expressed genes like *Igf2*, *Peg1* and *Peg3* promote nutrient transfer to the fetus, potentially at the expense of maternal resources [20, 21, 22, 23]. Furthermore, dysregulated expressions of several imprinted genes (e.g., *Igf2, Phlda2, H19* and *Gab1*) in the placenta have been implicated in various pregnancy complications, including preeclampsia, gestational diabetes and FGR [24, 25, 26, 27].

GAB1 belongs to a family of docking proteins that function as downstream scaffolding elements following activation of tyrosine kinase and cytokine receptors [28]. It plays a critical role in multiple processes of embryonic development by recruiting distinct effectors in various organs [28]. *Gab1* has been confirmed as a maternally imprinted gene in mice [29]. Genetic deficiency of *Gab1* in mice results in fetal demise in utero during mid‐gestation, characterised by placenta hypoplasia and defects in multiple fetal organs [30]. Transcriptomic study has revealed significant downregulation of *GAB1* expression in the placenta of FGR fetus [27]. However, the mechanisms through which GAB1 regulates placental development and its pathological role in FGR remain unclear.

In this study, we performed an in‐depth analysis by integrating single‐nucleus/single‐cell RNA sequencing data from human and mouse placentas along with transcriptomic data from FGR placenta. This approach allowed us to identify imprinted genes that may play a role in the dysregulation of maternal–fetal exchange, thereby affecting fetal growth. Using a food restriction (FR) mouse model and an in vitro trophoblast differentiation model, we investigated the functional mechanisms of GAB1 in response to nutrient stress and its modulation of trophoblast cell differentiation. Our findings indicate that nutritional restriction impairs GAB1 expression and protein stability through selective autophagic degradation, thereby hindering GAB1‐enhanced trophoblast syncytialisation via activation of MAPK signalling. These results highlight the crucial role of placental GAB1 in linking maternal nutrition status with fetal growth and provide potential therapeutic strategies for managing FGR.

## 2 Materials and Methods

### 2.1 Single‐Nucleus/Single‐Cell RNA Sequencing and Transcriptomic Data Analysis

The analysis of single‐nucleus/single‐cell RNA sequencing and transcriptomic data was conducted using publicly available datasets (GSE247038, GSE156125, GSE24129) [27, 31, 32]. The R language packages Seurat (version 4.3.0) [33] and Limma (version 3.62.1) were employed for these analyses. The R language package ggplot2 (version 3.5.1) [34] was used to generate volcano plots, uniform manifold approximation and projections (UMAPs), Venn diagrams, heatmaps and violin plots. The differentially expressed gene (DEG) dataset utilised for the creation of Venn diagrams is stored in a public repository (http://doi.org//10.6084/m9.figshare.28522598).

BigWig (bw) files containing genome‐wide enrichment of H3K18 acetylation (H3K18ac) in human placental trophoblast stem cells (hTSC) and STB‐D1 cells—a derivative of hTSC treated for 24 h with 2 μM forskolin (FSK), a PKA signaling agonist—were obtained from the GEO dataset GSE246278. The data were visualised using the Integrative Genomics Viewer (IGV) software.

### 2.2 Homologous Gene Mapping and Venn Diagram Analysis

Gene homology data between humans and mice were obtained from the Mouse Genome Informatics database (https://www.informatics.jax.org/homology.shtml). Human gene IDs were systematically mapped to their corresponding mouse homologues using established GENEID‐based annotation systems, enabling cross‐species comparative analysis through Venn diagram construction.

### 2.3 Pairwise Sequence Alignment

Protein sequences of GAB1 from various species, including *Danio rerio* (NP_001313642.1), *Rattus norvegicus* (NP_001101914.1), *Mus musculus* (NP_067331.2), *Macaca mulatta* (NP_001253361.1), *Bos taurus* (NP_001094671.1) and *Homo sapiens* (NP_002030.2), were obtained from the National Centre for Biotechnology Information (NCBI). The pairwise sequence alignment was conducted using the EMBOSS Water software (The EMBL‐EBI Job Dispatcher sequence analysis tools framework in 2024) available on the EMBL‐EBI web server (https://www.ebi.ac.uk/).

### 2.4 Food Restriction Mouse Model

All experiments involving mice were approved by the Animal Research Committee of the Institute of Zoology, Chinese Academy of Sciences. Virgin CD‐1 mice were housed in the specific pathogen‐free animal facility at the experimental animal centre of the Institute of Zoology, Chinese Academy of Sciences. The conditions were maintained at a humidity level of 35% ± 4%, a stable temperature of 24°C ± 1°C and a 12/12 h light/dark cycle. Virgin CD‐1 female mice (8–12 weeks old) were mated with fertile males (10 weeks old), and the day on which the vaginal plug was detected was recorded as embryonic Day E0.5. The pregnant mice were randomly divided into two groups: normal diet (CON, *n* = 10), FR diet (*n* = 10). The CON group continued to receive an *ad libitum* chow diet. The FR group was subjected to FR by providing 70% (wt) of their daily intake from E8.5. The mice were killed at E13.5, and the placenta tissues were collected.

### 2.5 RNA Extraction and Reverse Transcription‐Quantitative Polymerase Chain Reaction (RT‐qPCR)

Total RNA from tissues or cells was extracted using TRIzol reagent (Invitrogen) according to the manufacturer's instructions, and stored at −80°C. 2 μg of total RNA was reverse‐transcribed into cDNA in a total volume of 20 μL using 0.5 μg of oligo(dT) primer (GE Healthcare, CT, USA) and 500 units of Moloney murine leukaemia virus reverse transcriptase (New England Biolabs, Ontario, Canada). The reaction was carried out in a buffer (50 mM Tris–HCl (pH 8.3), 75 mM KCl, 3 mM MgCl_2_ and 10 mM dithiothreitol) containing 0.5 mM dNTPs for 2 h at 42°C. An aliquot of each cDNA sample (2 μL) was subjected to qPCR using a Lightcycler (Roche, Basel, Switzerland) in the presence of a reaction mixture containing SYBR Green PCR mix (Takara, Shiga, Japan), and 10 pmol of primers. The primer sequences for *M. musculus Gab1* were synthesised as follows: Forward primer: 5′‐GAAGTTGAAGCGTTATGCGTG‐3′; Reverse primer: 5′‐TCCAGGACATCCGGGTCTC‐3′. For the endogenous control gene *M. musculus Actb*: Forward primer: 5′‐GGCTGTATTCCCCTCCATCG‐3′; Reverse primer: 5′‐CCAGTTGGTAACAATGCCATGT‐3′. All PCR reactions were performed in triplicates, and the relative mRNA expression levels were determined using the 2^−ΔΔ*C*t^ method [35].

### 2.6 Western Blotting

The lysates from cultured cells or tissues were extracted using RIPA buffer (25 mM Tris–HCl, pH 7.4, 150 mM NaCl, 0.1% SDS, 1% NP40, 1 mM EDTA, 1 mM Na_3_VO_4_), supplemented with protease inhibitor cocktails (Sigma‐Aldrich). Following centrifugation (10,000g for 10 min) at 4°C, the supernatant was collected to remove insoluble fractions. The protein concentration was then measured using a BCA Protein Assay Kit (Beyotime Biotechnology), following the manufacturer's instructions. Cell lysates (30 μg) were subjected to 10% SDS‐PAGE and electrophoretic transfer to a nitrocellulose membrane (Millipore). The membranes were incubated at room temperature for 1 h in blocking buffer containing 5% BSA (Sigma), followed by incubation with primary antibodies in 5% BSA at 4°C, then incubated with horseradish peroxidase (HRP)‐conjugated secondary antibodies. The primary antibodies used are listed in Table S1. Signals were detected using ECL reagent (Amersham Life Science) and analysed with the GeneGnome XRQ Chemiluminescence Imaging System (Syngene) and Quantity One Analysis Software (version 4.4; Bio‐Rad). The relative density of each target protein was normalised based on the β‐actin level from the corresponding blot.

### 2.7 Immunofluorescence Staining and Confocal Microscopy

Immunofluorescence staining experiments were performed as previously described [36]. Tissues were freshly collected and embedded in optimal cutting temperature (OCT) compound (Sakura Finetek), followed by subjection to frozen section at 10 μm thickness. The frozen sections were briefly fixed in 4% paraformaldehyde (PFA) for 15 min, permeabilised in 0.1% Triton and blocked with 3% BSA, followed by incubation with primary antibodies (GAB1, CK7, MCT1 and MCT4, Table S1) in blocking buffer containing 2% BSA at 4°C overnight. The sections were further incubated with secondary antibodies including Alexa Fluor 488 goat antimouse IgG1 (A21121, Thermo Fisher Scientific), Alexa Fluor 568 goat antirabbit IgG (A11011, Thermo Fisher Scientific) or Alexa Fluor 647 goat antirabbit IgG (A21244, Thermo Fisher Scientific), along with DAPI (D9542, Sigma). The sections were imaged using a Zeiss LSM880 confocal microscope and ZEN Microscope Software.

### 2.8 Immunohistochemical Staining

Freshly collected tissues were fixed in 4% PFA, followed by standard dehydration and paraffin embedding, and sectioned at 5 μm thickness. The paraffin sections were dewaxed using a gradient of ethanol and rehydrated in PBS, then blocked with 3% BSA, followed by incubation overnight at 4°C with primary antibodies against GAB1 or laminin (Table S1). The sections were incubated with biotinylated horse antimouse or goat antirabbit secondary antibodies and visualised using diaminobenzidine (DAB) solution (Dako Cytomation, Glostrup, Denmark). To enhance contrast, the sections were counterstained with Carazzi's haematoxylin before mounting in glycerol/gelatin mounting medium (GG1‐10, Sigma). The tissue sections were imaged using a Zeiss Axiovert Z1 microscope coupled with Axiovision imaging software SE64 V4.8. The GAB1 intensity was analysed using ImageJ software (NIH). The ‘Smooth’ feature in ImageJ was employed after background subtraction and before threshold adjustments. The intensity of GAB1 was computed by the mean area of three randomly selected fields per slide, three slides per group [2].

### 2.9 Transmission Electron Microscopy (TEM)

A total of five pairs of placentas from FR and CON mice were included for the TEM experiment. Mouse placental tissues at 0.2 cm^3^ were freshly collected around the umbilical cord insertion site. The collected tissues were immediately immersed in 2.5% glutaraldehyde and 2% PFA in sodium cacodylate buffer, followed by fixation with 1% osmium tetroxide. Following routine dehydration, the tissues were embedded in epoxy resin. Ultrathin sections were mounted on 200 mesh grids and stained with uranyl acetate and lead citrate. The sections were observed using a JEM‐1400 TEM (JEOL).

### 2.10 Cell Culture and Treatment

The human choriocarcinoma trophoblastic cell line, BeWo, was purchased from the American Type Culture Collection. After thawing, these cells were cultured in a 1:1 mixture of Ham's F‐12 K (Kaighn's modification; Gibco) and Dulbecco's modified Eagle's medium (DMEM; Hyclone), supplemented with 10% fetal bovine serum (FBS; Hyclone) and sodium pyruvate [37]. To induce syncytialisation, BeWo cells were treated with FSK at 20 μM for 24–48 h.

To induce knockdown of GAB1, BeWo cells were seeded in 24‐well plates at a density of 20,000 cells per well and transiently transfected with 100 nM of specific siRNA targeting human GAB1 or nonspecific scramble siRNAs (Gene‐Pharma) as negative controls, using Lipofectamine 2000 (Thermo Scientific). Following 4 h of transfection, the medium was replaced with fresh F‐12 K/DMEM medium supplemented with 10% FBS and sodium pyruvate. The sequences of the siRNAs used in this study are listed in Table S2.

### 2.11 Plasmid Construction and Transfection

Full‐length human GAB1 cDNA (sequence obtained from the Human ORFeome Database, http://horfdb.dfci.harvard.edu/) was amplified via PCR and subcloned into the pcDNA4/myc‐His vector (Gab1^WT^). PCR‐based site‐directed mutagenesis plasmids were constructed using TransStart FastPFU DNA polymerase (TransGen Biotech, Beijing, China), employing the Gab1^WT^ plasmid as the template. Try^542^ and Leu^545^ of GAB1 were mutated to alanine to generate Gab1^lirMUT^. The sequences of the constructed plasmids were authenticated through sequencing. The primers used for plasmid construction are listed in Table S3.

Plasmid transfection experiments were performed as previously described [38]. Briefly, BeWo cells at 60%–70% confluency were treated with transfection media containing 1 μg specific plasmid mixed with Lipofectamine 2000 (Invitrogen, Shanghai, China). Fresh media were added following 24 h, and the cells were maintained before harvesting.

### 2.12 Calculation of Trophoblast Cell Fusion Index

Cells cultured in chamber slides were fixed with 4% PFA for 15 min, followed by permeabilisation with 0.1% Triton. After washing, the cells were blocked with 3% BSA and incubated with an antibody against E‐cadherin (#3195, CST, Table S1) at 4°C overnight. TRITC‐conjugated secondary antibody (ZSGB‐BIO, ZF‐0316) was added, along with DAPI (D9542, Sigma). Images were captured using a Leica confocal microscope (Leica Stellaris) and processed using LAS X software. The fusion index was determined by calculating the ratio of fused nuclei to the total nuclei within a given field of view. Eight distinct views per group were analysed for cell fusion index measurement.

### 2.13 Protein–Protein Interaction (PPI) Network Construction

The STRING database was used to predict potential interactions among the gene products of interest [39]. The PPI network diagram was constructed using the Cytoscape software (version 3.9.1; https://cytoscape.org/). Line thickness represents the strength of PPIs.

### 2.14 Statistical Analysis

All data were analysed using GraphPad Prism 10.4.1 statistical software (GraphPad Software). The results were presented as mean ± SD, derived from at least three independent experiments. Differences between groups were analysed using an independent sample *t* test or one‐way ANOVA, followed by post hoc Tukey–Kramer multiple comparison analysis. A *p* < 0.05 was deemed statistically significant.

## 3 Results

### 3.1 Integrative Data Analysis Highlights *GAB1* as a Potential Candidate That Links Maternal Nutritional Status With Fetal Growth Through Modulating Placental Villous Development

**FIGURE 1:**
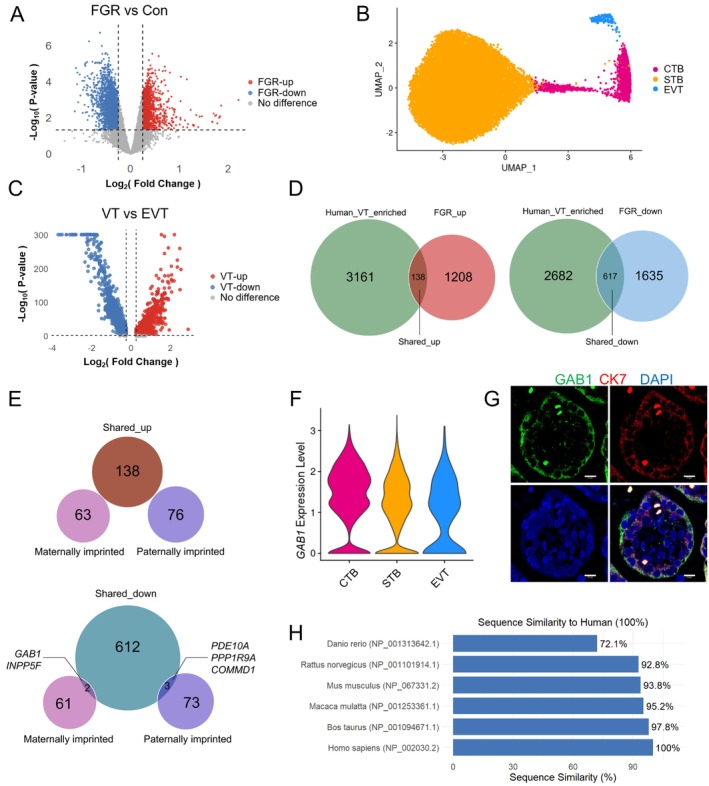
Integrated analysis of single‐nucleus RNA sequencing from human placentas and transcriptomic data from human FGR placentas to identify *GAB1* as a potential candidate that links maternal nutritional status with fetal growth through modulating placental villous development. (A) Volcano plot of gene expression in fetal growth restriction (FGR) versus gestational week matched control (Con). Red dots represent genes upregulated in FGR, blue dots represent genes downregulated in FGR. Thresholds were set at *p* < 0.05, |logFC| > 0.25. (B) Uniform manifold approximation and projection (UMAP) of single‐nucleus RNA sequencing data from human placentas. (C) Volcano plot of single‐nucleus RNA sequencing data from human placentas, comparing villous trophoblast lineage (VT) versus EVT. Red dots represent genes upregulated in the VT lineage, blue dots represent genes downregulated in the VT lineage. Thresholds were set at *p* < 0.05, |logFC| > 0.25. (D) Venn diagram showing intersection of genes enriched in the VT lineage and upregulated in FGR placentas (left); and enriched in the VT lineage and downregulated in FGR placentas (right). (E) Venn diagram of intersected genes from (D) with mouse maternally and paternally imprinted genes. (F) Violin plots show expression levels of *GAB1* in different subpopulations from single‐nucleus sequencing data in human placentas. (G) Immunofluorescence staining of GAB1 (green), cytokeratin 7 (CK7, red) and DAPI (blue) in human normal placenta. Scale bars, 10 μm. (H) Interspecies similarity scores for GAB1 protein sequences calculated using the BLOSUM62 matrix‐based algorithm.

Considering that FGR is frequently attributed to inadequate maternal nutrition delivery to the fetus, and given the placenta's critical role, particularly its villous tree structure, in modulating maternal–fetal nutrient allocation, we initiated an in‐depth analysis by integrating single‐cell/single‐nucleus RNA sequencing data from human and mouse placentas (GSE247038, GSE156125) [31, 32], along with transcriptomic data from FGR placenta (GSE24129) [27]. Principal component analysis (PCA) of the transcriptomic data from human FGR and control placentas demonstrated a distinct separation in the distribution of samples between the two groups (Figure S1). Subsequent DEG analysis identified 1346 upregulated genes and 2252 downregulated genes in FGR placenta compared to the control placenta, as illustrated in the volcano plot (Figure 1A).

Reanalysis of single‐nucleus RNA sequencing data from human placenta revealed that a total of 24,598 nuclei could be classified into three clusters based on the known marker genes, including villous CTB, STB and extravillous trophoblast (EVT) (Figure 1B). These clusters were further categorised into two distinct trophoblastic lineages: the villous trophoblasts (VT) lineage comprising CTB and STB, and the EVT lineage. DEG analysis indicated 3299 genes were enriched in the VT lineage compared with the EVT lineage, as shown in the volcano plot (Figure 1C).

Subsequently, we conducted a cross‐over analysis between the two databases. As illustrated in Figure 1D, 138 VT‐enriched genes were upregulated in FGR placenta (designated as shared_up genes), while 617 VT‐enriched genes were downregulated in FGR placenta (designated as shared_down genes) (Figure 1D). We further cross‐referenced these genes with the mouse imprinted gene database (https://www.geneimprint.com/). Notably, none of the 138 shared_up genes were imprinted, whereas the 617 shared_down genes included two maternally imprinted genes (*GAB1* and *INPP5F*) and three paternally imprinted genes (*PDE10A*, *PPP1R9A* and *COMMD1*) (Figure 1E).

Considering the conservation of the syncytialisation process between humans and mice, we conducted an analysis of single‐cell RNA sequencing data from mouse placentas at E9.5–E14.5 [31]. A total of 11,196 cells were identified and classified into six main clusters based on established marker genes. These clusters included labyrinth trophoblast progenitor cells (LaTP), STB layer I cells (SynT‐I), STB layer II cells (SynT‐II), trophoblast giant cells (TGCs), spongiotrophoblast cells (SpT) and glycogen trophoblast cells (Gly‐T) (Figure S1). Among these cell types, LaTP, SynT‐I and SynT‐II cells belong to the labyrinth lineage (LAB), which corresponds to the human VT lineage. DEG analysis revealed that 790 genes were specifically enriched in the LAB lineage compared to other trophoblast cell types, as shown in the volcano plot (Figure S1). Cross‐over analysis between the mouse LAB lineage and human FGR placenta identified 30 commonly upregulated genes and 124 commonly downregulated genes (Figure S1). Further cross‐referencing of these results with the mouse imprinted gene database (https://www.geneimprint.com/) showed that the 124 shared downregulated genes included two maternally imprinted genes, *Gab1* and *Peg10* (Figure S1).

This integrated data analysis identified *GAB1* as the sole common imprinted gene associated with FGR in both human and mouse placental villous lineage. Violin plots illustrated the enriched expression of the human *GAB1* gene in CTB and STB cells (Figure 1F), and the mouse *Gab1* gene in LaTP, SynT‐1 and SynT‐II cells (Figure S1). Immunofluorescent staining of the human placenta confirmed the predominant localisation of GAB1 in VT (Figure 1G). Furthermore, sequence similarity analysis revealed high conservation (> 90%) of the *GAB1* gene sequence among mammals (Figure 1H).

Collectively, these findings suggest that *GAB1* may serve as a potential candidate that links maternal nutritional status with fetal growth through modulating placental villous development.

### 3.2 Maternal Food Restriction During Pregnancy in Mice Leads to Fetal Growth Retardation, Impaired Development of the Placental Labyrinth and Decreased Expression of GAB1

**FIGURE 2:**
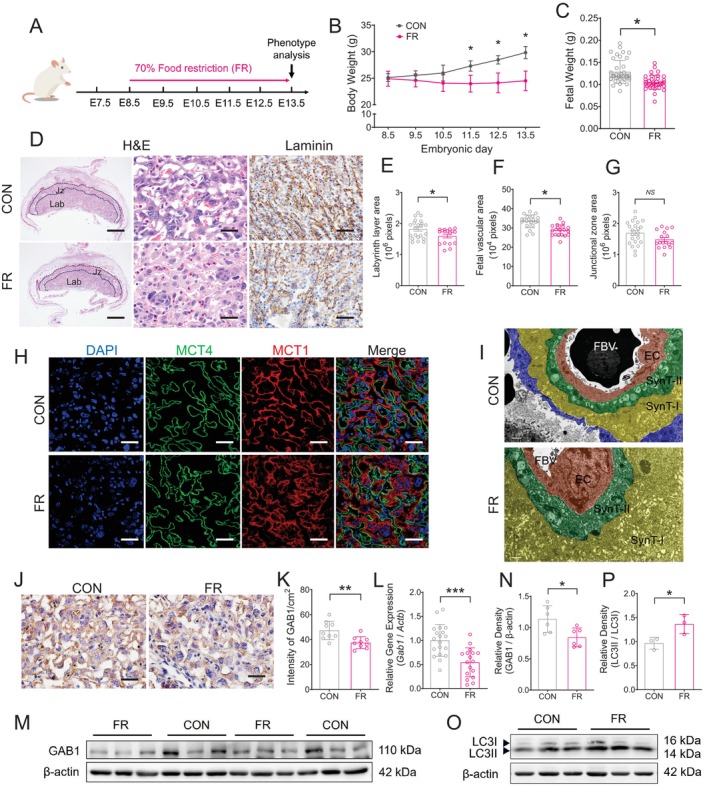
Maternal food restriction in pregnant mice resulted in abnormal development of the placental labyrinth layer and downregulation of GAB1 expression. (A) A schematic depiction of the experimental setup. Control group (CON) pregnant CD‐1 mice were given an *ad libitum* chow diet, while the food restriction group (FR) was subjected to a diet comprising 70% (wt) of their daily intake from gestational Day 8.5. The mice were euthanised at E13.5, and phenotypes were analysed. (B and C) Net maternal weight (excluding foetuses) from E8.5 to E13.5 (B) and fetal weight at E13.5 (C) in mice from CON and FR groups. (D–G) Haematoxylin–eosin (H&E) and immunohistochemistry (IHC) staining against laminin (D), and the statistical analysis of the labyrinth layer area (E), fetal vascular area (F) and junctional zone area (G) in the mouse placentas from CON and FR groups. Lab, labyrinth zone. Jz, junctional zone. Scale bars in (D) indicate 500 μm in the left panels, 20 μm in the middle panels and 50 μm in the right panels. (H) Immunofluorescence staining against MCT4 (green) and MCT1 (red) in the mouse placentas from CON and FR groups. Scale bars indicate 20 μm. (I) Representative transmission electron microscopy (TEM) images of the labyrinth layer in the mouse placentas from CON and FR groups. FBV, fetal blood vessels; EC, fetal vascular endothelial cells; SynT‐II, the second layer of syncytiotrophoblast; SynT‐I, the first layer of syncytiotrophoblast. Scale bars indicate 1 μm. (J and K) IHC staining against GAB1 (J) and statistical analysis (K) in the mouse placentas from CON (*n* = 9) and FR (*n* = 9) groups. Scale bars, 20 μm. (L) mRNA level of *Gab1* in the mouse placentas from CON (*n* = 19) and FR (*n* = 17) groups. (M and N) Western blots (M) and corresponding quantification (N) for GAB1 in the placentas of CON (*n* = 6) and FR (*n* = 6) groups. (O–P) Western blots (O) and corresponding quantification (P) for LC3 in the placentas of CON (*n* = 3) and FR (*n* = 3) groups. Data were shown as mean ± SD, and the analysis was conducted using a two‐tailed *t* test. *p < 0.05, **p < 0.01, ***p < 0.001.

To investigate the link between placental GAB1 and maternal nutritional status, we established a FR mouse model wherein pregnant mice were subjected to a diet comprising 70% of their normal daily intake from embryonic Days E8.5–E13.5. Fetal and placental outcomes were evaluated at E13.5 (Figure 2A). This dietary regimen resulted in a significant reduction in maternal weight (Figure 2B) and restricted fetal growth (Figure 2C). Histological analysis and laminin staining revealed markedly impaired development of the placental labyrinth in FR mice, as evidenced by a significant decrease in the labyrinth area, compromised fetal vessel branching and reduced fetal vascular area within the labyrinth layer (Figure 2D–F). The development of the placental junctional zone was not significantly affected (Figure 2D,G). In the mouse placenta, two closely adjacent layers of STBs, SynT‐I and SynT‐II, form a fetal‐maternal barrier in the labyrinth layer, which can be separately marked by monocarboxylate transporter 1 (MCT1) and MCT4 [2]. Immunofluorescent staining for MCT1 and MCT4 in the mouse placenta demonstrated disorganised alignment of the syncytial layers as well as compromised fetal blood vessels in the FR group compared with the control group (Figure 2H). TEM of the placentas showed dramatic thickening of the two syncytial layers in the placenta of the FR group (Figure 2I). These phenotypes in FR mice indicate that maternal nutritional restriction leads to impaired placenta development and fetal growth retardation.

Immunohistochemistry, quantitative RT‐PCR and Western blot analyses revealed a significant decrease in GAB1 expression within the placenta of FR mice, particularly in the labyrinth trophoblasts (Figure 2J–N). Given that nutrient restriction can induce autophagy in various cell types, we evaluated LC3 expression in the placenta of FR mice. As shown in Figure 2O,P, the ratio of LC3II to LC3I was significantly elevated in the placentas of the FR group compared to the control group, indicating an increase in autophagic activity in the placentas under maternal nutrient stress.

Collectively, these findings suggest that maternal FR during pregnancy leads to fetal growth retardation, which is associated with impaired development of the placental labyrinth and reduced placental expression of GAB1.

### 3.3 Nutritional Deprivation in Trophoblasts Induces Autophagic Degradation of GAB1

**FIGURE 3:**
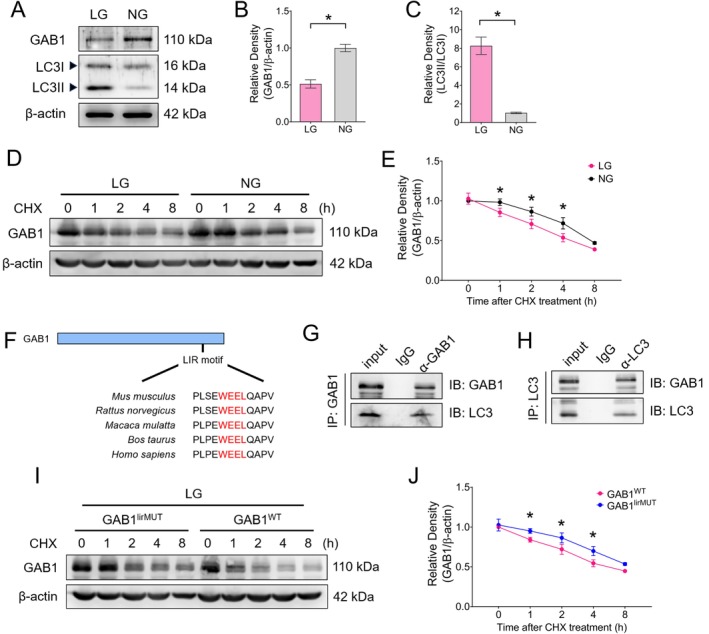
Glucose deprivation in human trophoblasts induces autophagic degradation of GAB1. (A–C) Western blots (A) and corresponding quantification (B and C) of GAB1 and LC3 in BeWo cells cultured in normal glucose (NG, 4.5 g/L glucose) or low glucose (LG, 1 g/L glucose) medium. (D and E) Typical result of Western blots (D) and statistical analysis (E) for protein stability analysis of GAB1 in BeWo cells cultured in NG or LG condition. (F) Schematic depiction of the conservation of the LC3‐interacting regions (LIR) region (Red) among mammals. (G and H) Co‐immunoprecipitation analysis showing the binding between GAB1 and LC3 in BeWo cells. (I and J) Typical result of Western blots (I) and statistical analysis (J) for protein stability analysis of GAB1 in BeWo cells that were transfected with plasmid carrying wild type GAB1 cDNA (GAB1^WT^) or cDNA with mutated LIR regions in GAB1 (GAB1^lirMUT^), maintained in LG media. Data were shown as mean ± SD, and the analysis was conducted using a two‐tailed *t* test. *p < 0.05.

To investigate the association between the downregulation of placental GAB1 under nutrient restriction and the autophagy process, we conducted in vitro experiments using BeWo cells, a human trophoblastic cell line with syncytialisation capability [2, 40]. Cells were cultured for 24 h in media supplemented with either normal glucose concentration (NG, 4.5 g/L) or low glucose concentration (LG, 1 g/L). Our findings revealed that GAB1 expression was evidently reduced in the LG group compared to the NG group, accompanied by an elevated LC3II/LC3I level (Figure 3A–C). Protein stability assays revealed that the LG condition accelerated GAB1 protein degradation (Figure 3D,E). To determine whether GAB1 can be degraded via autophagy, we performed protein sequence analysis and identified a highly conserved LIR motif in the GAB1 sequence across mammalian species (Figure 3F), which mediates direct binding to autophagosomal LC3 [41]. Co‐immunoprecipitation experiments in BeWo cells confirmed the direct protein binding between GAB1 and LC3 (Figure 3G,H). When we introduced site mutations in the LIR motif of GAB1, the LG‐induced GAB1 degradation in BeWo cells was markedly attenuated (Figure 3I,J). These results suggest that GAB1 in placental trophoblast cells undergoes selective autophagic degradation under nutrient‐restricted conditions.

### 3.4 GAB1 Enhances Trophoblast Syncytialisation Through Activation of MAPK Signal

**FIGURE 4:**
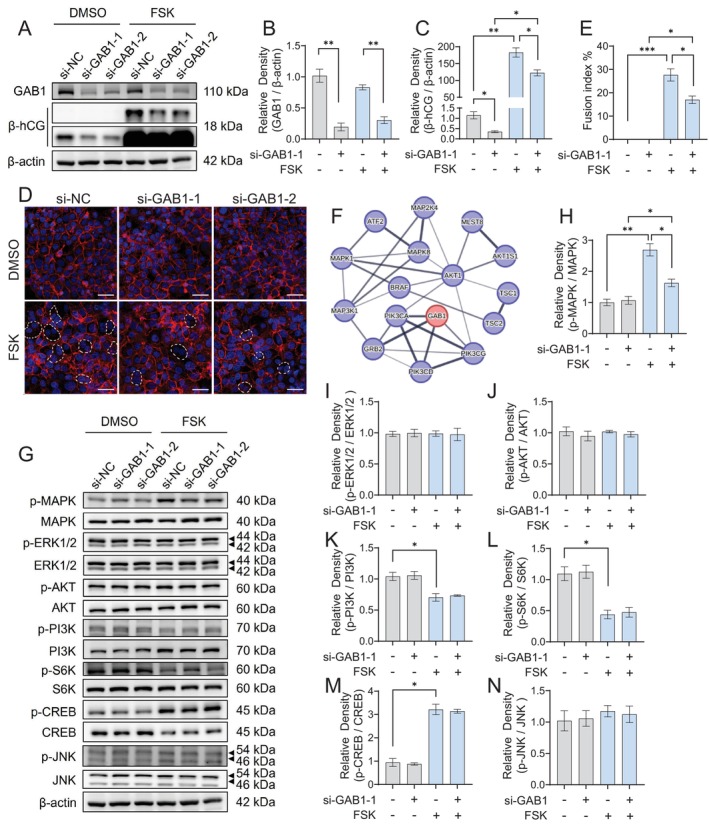
GAB1 knockdown inhibited trophoblast syncytialisation and the activation of MAPK signal. (A–C) Western blots (A) and statistical analysis of GAB1 (B), β‐hCG (C) in BeWo cells transfected with GAB1‐specific siRNA (si‐GAB1‐1 and si‐GAB1‐2) or nonspecific control siRNA (si‐NC), together with or without 20 μM forskolin (FSK) treatment. (D and E) Representative immunostaining of E‐cadherin (D) and the statistical analysis results of cell fusion index in BeWo cells with GAB1 knockdown and FSK treatment. Scale bars indicate 50 μm. (F) A PPI network constructed using STRING to identify key interaction pathways with GAB1. (G–N) Western blots (G) and statistical analysis results of p‐MAPK/MAPK (H), p‐ERK1/2 / ERK1/2 (I), p‐AKT/AKT (J), p‐PI3K/PI3K (K), p‐S6K/S6K (L), p‐CREB/CREB (M) and p‐JNK/JNK (N) in BeWo cells with GAB1 knockdown and FSK treatment. (A) and (G) depict representative Western blotting results from the same batch of samples. Data were shown as mean ± SD, and the analysis was conducted using one‐way ANOVA, followed by post hoc Tukey–Kramer multiple comparison analysis. *p < 0.05, **p < 0.01, ***p < 0.001.

Based on the observed phenotypes of placental development in FR mice, we further investigated the role of GAB1 in regulating trophoblast cell differentiation toward the syncytial lineage. An in vitro model using BeWo cells was employed to induce syncytialisation by FSK treatment [2, 40]. The expression levels of β‐hCG and the cell fusion index were quantified to assess the degree of syncytialisation. Our findings revealed that specific siRNA‐mediated knockdown of GAB1 in BeWo cells resulted in a marked reduction of β‐hCG expression and a decrease in the cell fusion index following FSK stimulation (Figure 4A–E). These results suggest that GAB1 plays a direct role in modulating trophoblast syncytialisation.

Given that GAB1 functions as a docking protein with potential interactions with multiple signalling proteins, we further explored its downstream signalling pathways involved in trophoblast syncytialisation. PPI analysis indicated that GAB1 can interact with MAPK, PI3K, AKT, mTOR, ERK and CREB signalling pathways (Figure 4F). We evaluated the activation of these signalling pathways in BeWo cells upon FSK stimulation, both in the presence and absence of GAB1 knockdown, and found that GAB1 knockdown significantly attenuated the activation of the MAPK pathway in syncytialised trophoblast cells, while other pathways remained unaffected (Figure 4G–N).

**FIGURE 5:**
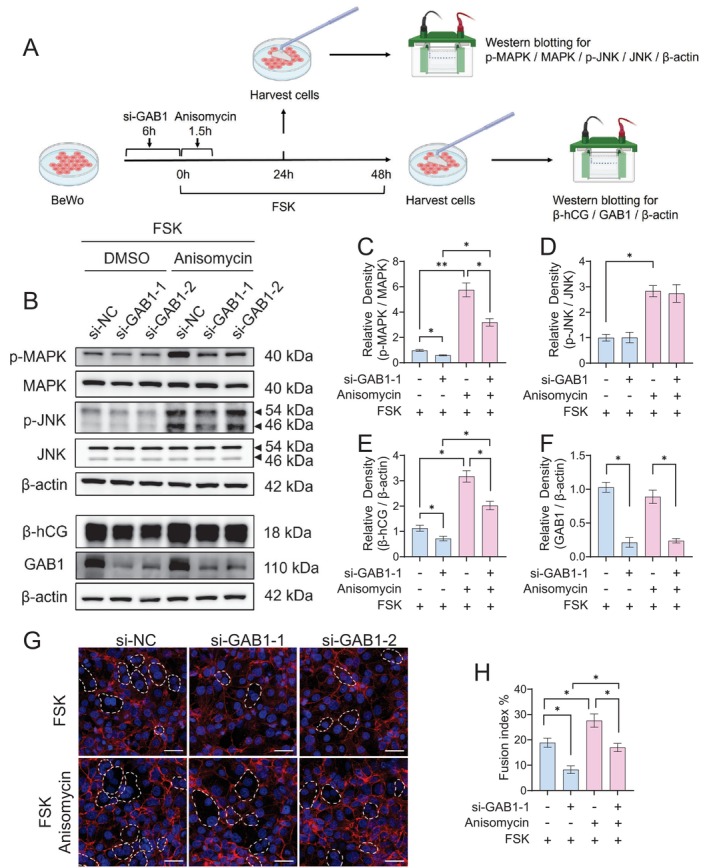
GAB1 promotes trophoblast syncytialisation via the MAPK signalling pathway. (A) Schematic depiction of the experimental setup. BeWo cells that were transfected with GAB1‐specific siRNA or si‐NC were treated with 20 μM FSK for 48 h, together with a 1.5 h‐duration of 1 μM anisomycin treatment. (B–F) Western blotting (B) and statistical analysis results of p‐MAPK/MAPK (C), p‐JNK/JNK (D), β‐hCG (E) and GAB1 (F) in BeWo cells with various treatments as indicated. (G and H) Representative immunostaining of E‐cadherin (G) and the statistical analysis results of cell fusion index (H) in BeWo cells with various treatment as indicated. Scale bars, 50 μm. Data were shown as mean ± SD, and the analysis was conducted using one‐way ANOVA, followed by post hoc Tukey–Kramer multiple comparison analysis. *p < 0.05, **p < 0.01. The representation shown in (A) was created with BioRender.com.

To further investigate whether the enhancing effect of GAB1 on trophoblast syncytialisation is directly mediated by the MAPK pathway, BeWo cells with GAB1 knockdown were treated with a specific MAPK/JNK activator, anisomycin [42]. The level of cell syncytialisation was assessed under FSK stimulation (Figure 5A). As illustrated in Figure 5, short‐term treatment with anisomycin effectively activated the MAPK and JNK pathways in BeWo cells (Figure 5B–D), leading to a significant restoration of β‐hCG expression and the cell fusion index that had been suppressed by GAB1 knockdown (Figure 5B,E,G,H). Notably, anisomycin treatment did not affect GAB1 expression (Figure 5B,F).

Collectively, these findings suggest that GAB1 enhances trophoblast syncytialisation via activation of the MAPK signalling pathway.

## 4 Discussion

**FIGURE 6:**
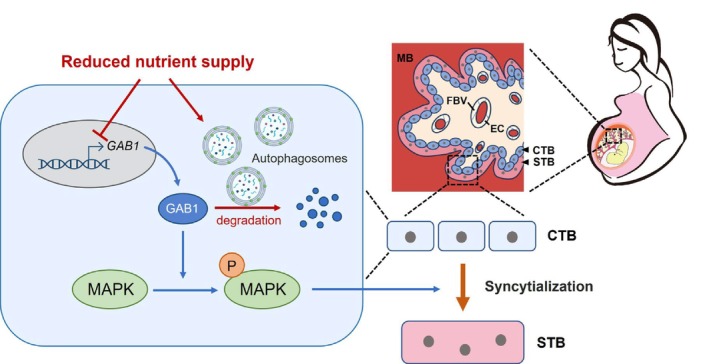
A conceptual model illustrating the mechanism by which maternal nutritional restriction influences fetal development through governing GAB1 expression and stability in the placenta. GAB1 functions in the placenta to promote trophoblast syncytialisation via activation of the MAPK signalling pathway. Under conditions of restricted nutrient supply, both the gene expression and protein stability of GAB1 in placental trophoblasts are reduced, with the latter occurring through LIR motif‐mediated selective autophagic degradation. Consequently, GAB1‐mediated trophoblast syncytialisation is impaired, leading to compromised formation of the placental unit responsible for maternal–fetal exchange. The schematic diagram was created with BioRender.com. CTB, cytotrophoblast; EC, fetal vascular endothelial cells; FBV, fetal blood vessels; MB, maternal blood sinus; STB, syncytiotrophoblast.

Appropriate maternal–fetal nutrient allocation significantly influences the health of both the mother and fetus during mammalian pregnancy, particularly under conditions of limited nutrient availability [43]. The placenta serves as a critical organ that mediates the conflict between the two entities [2]. FGR is a typical complication arising from insufficient or disrupted maternal nutritional conditions, leading to inadequate fulfilment of fetal demands [5, 6, 7]. This study underscores the pivotal role of placental GAB1 in linking maternal nutrition deprivation with FGR. Our integrated analysis of single‐nucleus/single‐cell RNA sequencing data from human and mouse placentas, transcriptomic data for FGR placenta and an imprinted gene database identified *GAB1* as the sole imprinted gene enriched in the placental villous lineage and dysregulated in FGR placentas. Combined with experiments utilising both in vivo mouse model and in vitro trophoblast cell differentiation models, it is evident that the placenta responds to persistent maternal nutrient stress and downregulates GAB1 levels, resulting in reduced trophoblast syncytialisation and consequently impaired maternal–fetal material exchange and retarded fetal growth. The downregulation of GAB1 manifests through distinct mechanisms at different levels: transcriptional regulation at the mRNA level and posttranslational modification via selective autophagic degradation at the protein level (Figure 6). These findings provide a molecular mechanism that modulates maternal–fetal nutrient allocation via the placenta.

A growing body of evidence, primarily derived from studies in mice, has demonstrated that imprinted genes play a crucial role in regulating nutrient allocation during pregnancy by influencing placental development and function [21, 22]. For instance, the deletion of maternally expressed genes such as *H19, Igf2r* and *Phlda2* results in placental and fetal overgrowth [21, 23, 44]. Conversely, the deletion of paternally expressed genes like *Igf2*, *Peg1* and *Peg3* leads to a smaller placenta size, which is associated with reduced fetal growth [20, 21, 22, 23]. Dysregulation of imprinted genes, including *Igf2, Phlda2, H19* and *Gab1* has been observed in placentas affected by pregnancy complications including preeclampsia, gestational diabetes and FGR [24, 25, 26, 27]. The mechanisms by which *Igf2* and *Igf2r* regulate nutrient allocation during pregnancy have been extensively studied using various genetic mouse models. Specifically, *Igf2* is involved in controlling the formation of placental microvasculature [45], regulating the expression of glucose and amino acid transporters by the placenta [46, 47], and modulating placental hormone production which facilitates maternal insulin resistance, thereby limiting glucose utilisation by maternal tissues and promoting increased glucose transport to the fetus [20]. Our findings, along with those of others, reveal that GAB1 functions differently from IGF2. Deficiency of the *Gab1* gene in mice results in embryonic lethality associated with significantly hypoplasia of placental labyrinth trophoblasts, which undergo syncytial differentiation to form maternal–fetal exchange unit, while junctional zone trophoblasts responsible for placental hormone production remain unaffected [30]. In this study, GAB1 downregulation in food‐restriction mice is predominantly associated with the disorganisation of labyrinth trophoblasts rather than disorders in the junctional zone. In vitro experiments using human trophoblast cells further demonstrate that GAB1 enhances trophoblast syncytialisation through MAPK signalling pathway. Consequently, GAB1 likely plays a critical role in modulating trophoblast syncytial differentiation to establish villous structure essential for maternal–fetal nutrient exchange. Under conditions of severe maternal malnutrition, as evidenced by the suppressed weight gain in dams in our FR mice model, the downregulation of GAB1 may serve to limit material delivery to the fetus, thereby prioritising maternal health [48].

The adaptive regulation of the placenta in response to restricted maternal nutrient availability is a complex process. Our recent studies have demonstrated that amino acid shortage induces macropinocytotic activity in STBs, mediated by the mTOR‐TFEB signalling pathway [1, 2]. Specifically, amino acid scarcity suppresses mTOR signalling in STB, thereby activating their ability to utilise pinocytosed extracellular proteins as an alternative source of amino acids [2]. This mechanism represents a flexible strategy employed by the placenta to efficiently adapt to fluctuations in amino acids and ensure a stable supply of biosynthetic substrates to the growing fetus. However, the activation of macropinocytosis in the placenta is not significant under conditions of restricted glucose or fatty acid supply (data not shown). Conversely, our most recent investigation into the metabolic programming and cell fate regulation of hTSC reveals that basal glucose–acetate metabolism is essential for hTSC syncytialisation by maintaining histone acetylation. A brief deficiency in glycolysis leads to a significant reduction in syncytialisation potential [49]. Consistent with these findings, LG supply impairs syncytium formation in BeWo cells, at least partially due to autophagic degradation of GAB1. Furthermore, analysis of our database on glucose–acetate‐driven histone acetylation (GSE246278) [49] indicates the potential regulation of GAB1 expression by H3K18ac (Figure S2), further highlighting the sensitivity of this gene to glucose metabolism. These findings underscore the intricate and sophisticated regulation of maternal–fetal nutrient allocation by the placenta, emphasising the need for detailed investigation into the pathogenesis of FGR.

A notable issue is the frequent inconsistencies observed between mice and humans regarding the imprinting status of placental genes. For instance, placenta‐specific imprinting at the *KCNQ1OT1* loci is not conserved in humans due to the absence of allelic repressive histone modifications [48]. Additionally, *IGF2R* and *SLC22A2* exhibit polymorphic imprinting patterns in humans [50]. Furthermore, while *Sfmbt2* and *Gab1* are paternally expressed in the mouse placenta, their human orthologues are biallelically expressed [29, 51]. Moreover, most placenta‐specific imprinted regions identified through genome‐wide screens for differentially methylated regions (DMRs) in humans do not show differential methylation in the mouse placenta, indicating widespread differences in imprinting during evolution [52, 53]. The mechanisms underlying these evolutionary differences in gene imprinting status remain elusive. Nonetheless, the function of GAB1 in enhancing placental and fetal development appears to be conserved between humans and mice, as evidenced by our in vivo and in vitro findings as well as studies in genetically manipulated mice.

In our current study, we propose that nutrient deficiency leads to the suppression of GAB1 at both mRNA and protein levels, with the latter being primarily through autophagic degradation. It remains unclear whether ubiquitination degradation of GAB1 is also involved during this process. Studies in the human gastric carcinoma cell line GTL‐16 cells demonstrated that prolonged HGF/SF‐MET signalling led to both multiubiquitination (proteasomal targeting) and K63‐linked polyubiquitination (lysosomal routing) of GAB1 in a CBL‐dependent manner [54]. This dual ubiquitination mechanism regulated GAB1 stability and downstream MAPK/ERK signalling, suggesting a possible convergence of autophagy and ubiquitin‐mediated degradation pathways. So far, there has been scarce evidence regarding the posttranslational modification of GAB1 under dietary restriction. Our present study demonstrated that nutrient stress may lead to GAB1 degradation involving autophagy, whereas further mechanistic studies are warranted to dissect the interplay between autophagic degradation and ubiquitination by evaluating ubiquitination markers (e.g., K48‐ vs. K63‐linked chains) and autophagy‐ubiquitination crosstalk regulators (e.g., p62/SQSTM1) in nutrient‐deprived models.

This study has certain limitations. First, although we demonstrated the regulation of trophoblast syncytialisation by the GAB1‐MAPK cascade, it remains unresolved how GAB1 regulates the phosphorylation of MAPK. Evidence from the study of cytokine signalling provides some valuable hints. Upon IL‐6 stimulation in normal human dermal fibroblasts (NHDF) and HEK293 cells, MAPK pathway activation occurs in two phases: an initial GAB1‐independent phase driven by SHP2 binding to the receptor complex, followed by a GAB1‐dependent amplification phase which necessitates the coordinated recruitment of Grb2 and SHP2 to GAB1 [55]. Considering the characteristics of GAB1 as a docking protein, we propose that GAB1 may function through the recruitment of Grb2, SHP2 or other kinases to activate MAPK and regulate trophoblast syncytialisation. More experiments are needed to address this hypothesis. Second, our findings demonstrate the activation of MAPK by GAB1 to enhance trophoblast syncytialisation; however, the downstream targets of GAB1‐MAPK signalling remain to be identified. As a docking protein, GAB1 has been shown to interact with multiple signalling pathways, as illustrated by the PPI analysis conducted in the present study (Figure 4F). Further experiments using the induced syncytialisation cell models revealed that MAPK signalling is the predominant downstream pathway mediating the syncytium‐enhancing effect of GAB1. Although PKA‐induced CREB has been recognised as an essential transcription factor for trophoblast syncytialisation [56, 57, 58], GAB1 does not influence CREB activation. The importance of the MAPK signalling pathway in regulating syncytial differentiation has been highlighted by the studies involving in‐depth RNA sequencing analysis, genome‐scale DNA methylation and ChIP‐seq analyses [59, 60]. Substantial upregulation of genes associated with the MAPK signalling pathway during trophoblast syncytialisation includes FOS, MYC, NR4A1 and several members of the DUSP family and FGF family [59]. Further investigation is warranted to explore the downstream effectors of GAB1‐MAPK signalling in promoting trophoblast syncytialisation and to examine the rescue effect on fetal growth by targeting these effectors in FR mice, which may provide new therapeutic strategies for intervening in FGR. Third, the partial rescue of GAB1 knockdown‐induced reduction in syncytialisation efficiency upon MAPK reactivation (Figure 5B–H) suggests that there may exist additional GAB1‐dependent mechanisms beyond MAPK phosphorylation. Further investigation on the phosphoproteomic profiling to map GAB1‐dependent kinase‐substrate networks will be helpful to systematically reveal the mechanisms underlying GAB1‐regulated trophoblast differentiation.

In summary, we performed an extensive data integration analysis complemented by in vivo and in vitro experiments using a food‐restriction mouse model and trophoblast differentiation models. Our results demonstrate that nutritional restriction compromises placental GAB1 expression and protein stability via autophagic degradation, consequently impeding GAB1‐MAPK signalling‐mediated trophoblast syncytialisation. These findings underscore the pivotal role of placental GAB1 in linking maternal nutritional status with fetal growth, and offer potential therapeutic avenues for managing FGR.

## Author Contributions

Mingming Fan, Hongyu Wu and Ming Liu conducted experiments, analysed the data and drafted the manuscript. Yuan Xie, Xin Yu and Feiyang Wang performed transcriptomic data analysis. Zhenyu Xiao and Hongmei Wang assisted with data interpretation. Yan‐Ling Wang and Xuan Shao conceived and supervised the study and coordinated the research team. All authors reviewed the final manuscript without dissent before submission.

## Ethics Statement

The study protocol was approved by the Ethics Committee at the Institute of Zoology, Chinese Academy of Sciences.

## Conflicts of Interest

The authors declare no conflicts of interest.

## Supporting information


**Data S1.** Supporting Information.

## Data Availability

The data that support the findings of this study are available from the corresponding author upon reasonable request.
