# Supplementary material for: Maternal Nutritional Status Governs Fetal Development by Modulating Imprinting Gene GAB1‐Mediated Trophoblast Differentiation in the Placenta
Source: Cell Prolif. 2025 Jun 23;58(12):e70069. doi: 10.1111/cpr.70069 (PMC12686128; doi:10.1111/cpr.70069)
Supplement: Supplementary file 1 — Data S1. Supporting Information. [file CPR-58-e70069-s001.docx]

**Supplemental Material for**

**Maternal Nutritional Status Governs Fetal Development by Modulating Imprinting Gene GAB1-Mediated Trophoblast Differentiation in the Placenta**

**Mingming Fan^1,3†^, Hongyu Wu^1,3†^, Yuan Xie^1,3†^, Ming Liu^4^, Xin Yu^1,2^, Feiyang Wang^1,2^, Zhenyu Xiao^2,5^, Hongmei Wang^1,2,3^, Xuan Shao^1,2,3*^, Yan-Ling Wang^1,2,3*^**

**^*^ Corresponding authors:**

Yan-Ling Wang

wangyl@ioz.ac.cn

Xuan Shao

shaoxuan@ioz.ac.cn

^†^ These authors contribute equally to this work.

**This file includes:**

Figures S1 to S2

Tables S1 to S3


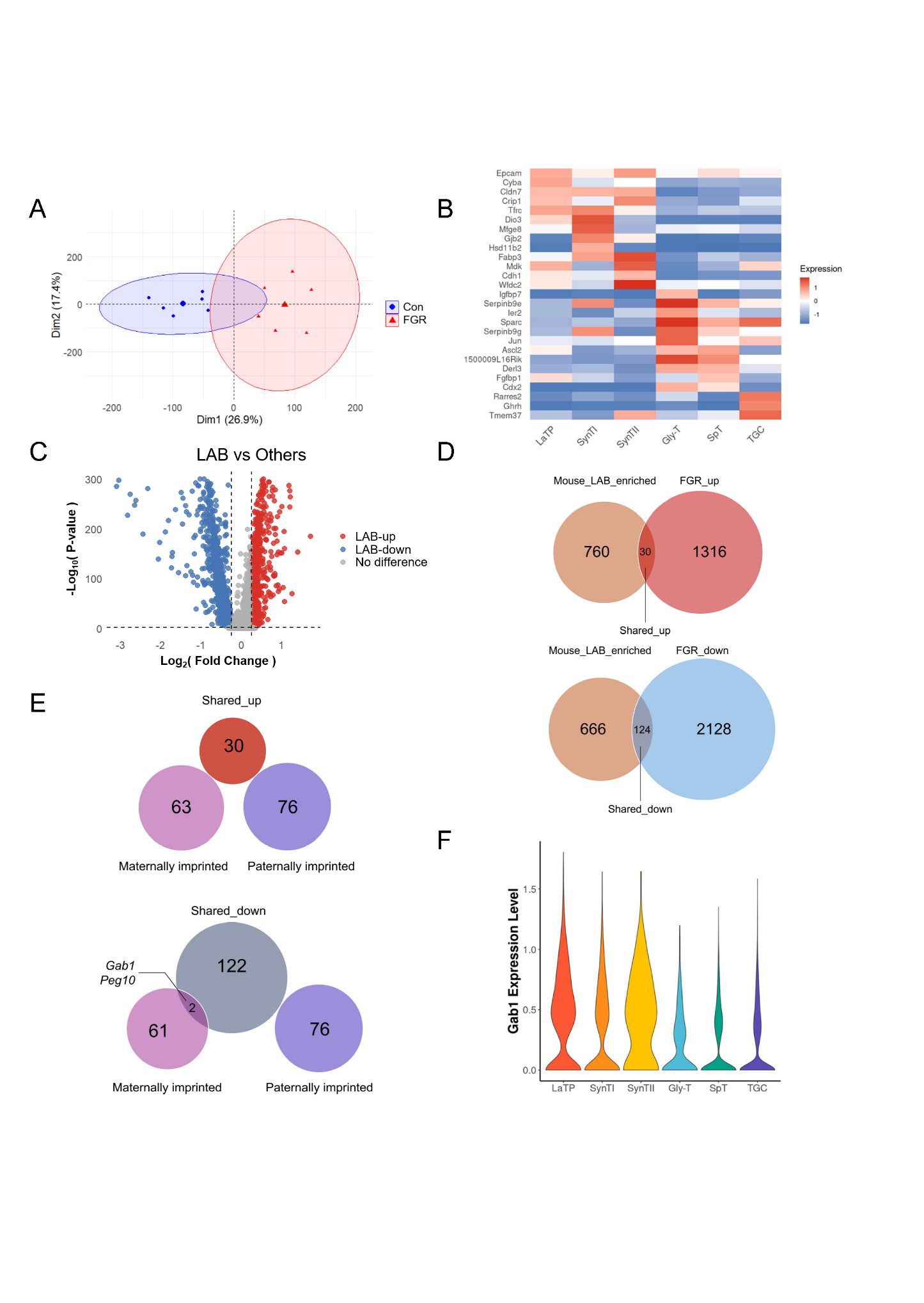


**Figure S1.** Integrated analysis of single-cell RNA sequencing from mouse placentas and transcriptomic data from human FGR placentas to identify *Gab1* as a potential candidate that links maternal nutritional status with fetal growth through modulating placental labyrinth development. (A) Principal component analysis (PCA) of the transcriptomic data analysis from human FGR (FGR) and control placentas (Con) demonstrated a clear differentiation in the distribution of samples between the two groups. (B) Heatmap showing the relative expression of the top marker genes defining the six trophoblast subsets. The top expressed genes for each subset were defined using non-parametric two-sided Wilcoxon rank sum test in Seurat. (C) Volcano plot of single-cell RNA sequencing data from mouse placentas, comparing the labyrinth lineage (LAB) (LaTP, SynT-I, and SynT-II) vs Others (TGC, Gly-T, SpT). Red dots represent genes upregulated in the LAB lineage; blue dots represent genes downregulated in the LAB lineage. Thresholds were set at p < 0.05, |logFC| > 0.25. (D) Venn diagram showing intersection of genes enriched in the LAB lineage and upregulated in FGR placentas (upper); and enriched in the LAB lineage and downregulated in FGR placentas (lower). (E) Venn diagram of intersected genes from (D) with mouse maternally and paternally imprinted genes. (F) Violin plots show expression levels of *Gab1* in different subpopulations from single-cell RNA sequencing data in mice placentas.


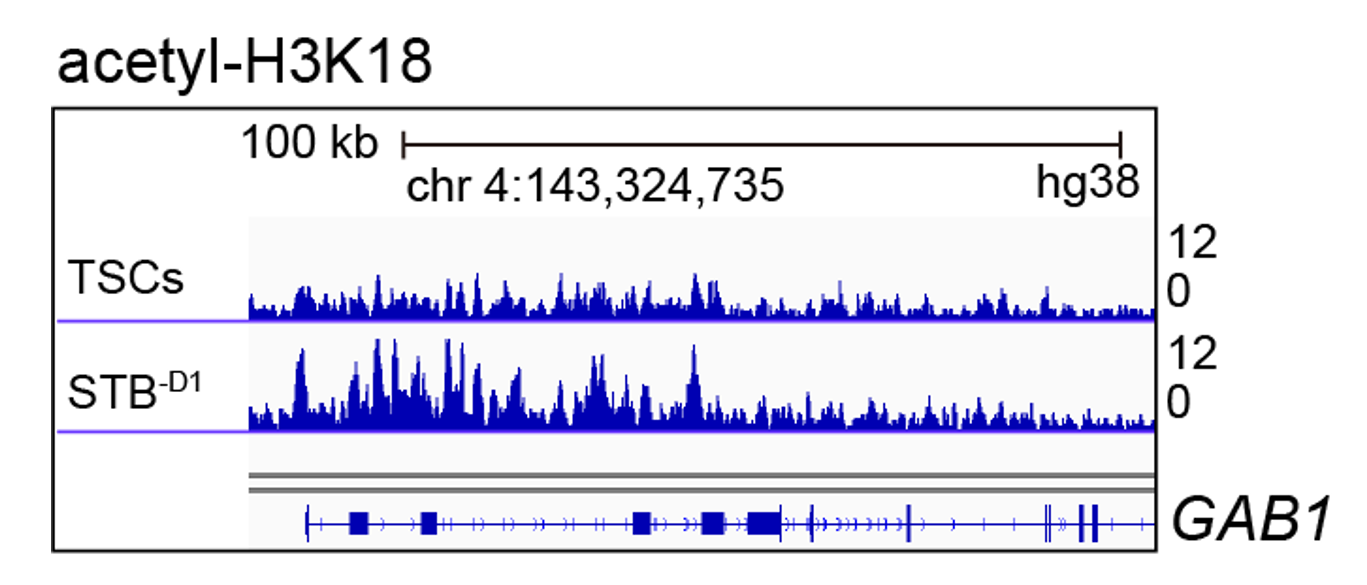


**Figure S2.** IGV visualization of H3K18 acetylation (H3K18ac) enrichment at the transcription start site (TSS) region of the *GAB1* gene in human placental trophoblast stem cells (hTSC) and STB-D1 cells (hTSC cells treated with forskolin at 2 μM for 24 h). The data was obtained from CUT&Tag (cleavage under targets and tagmentation) profiling of H3K18ac (GSE246278).

| **Table S1. List of primary antibodies used in this study** | | | |  |  |
| --- | --- | --- | --- | --- | --- |
| Peptide/protein target | Manufacturer, catalog # | Species | RRID | | Dilution used |
| MCT1 | sc-365501 | Mouse | AB_10841766 | | 1:500 |
| MCT4 | 22787-1-AP | Rabbit | AB_11182479 | | 1:500 |
| GAB1 | ab59362 | Rabbit | AB_941700 | | 1:1000 |
| LC3B | L7543 | Rabbit | AB_796155 | | 1:1000 |
| MAPK | #8690 | Rabbit | AB_10999090 | | 1:1000 |
| Phospho-MAPK (Thr180/Tyr182) | #4511 | Rabbit | AB_2139682 | | 1:1000 |
| PI3 Kinase p85 | #4257 | Rabbit | AB_659889 | | 1:1000 |
| Phospho-PI3 Kinase p85 (Tyr458)/p55 (Tyr199) | #4228 | Rabbit | AB_659940 | | 1:1000 |
| Akt | #9272 | Rabbit | AB_329827 | | 1:1000 |
| Phospho-Akt (Ser473) | #9271 | Rabbit | AB_329825 | | 1:1000 |
| p70 S6 Kinase | #2708 | Rabbit | AB_390722 | | 1:1000 |
| Phospho-p70 S6 Kinase (Ser371) | #9208 | Rabbit | AB_330990 | | 1:1000 |
| ERK1/2 | #4695 | Rabbit | AB_390779 | | 1:1000 |
| Phospho-ERK1/2 (Thr202/Tyr204) | #4370 | Rabbit | AB_2315112 | | 1:2000 |
| CREB | #9197 | Rabbit | AB_331277 | | 1:1000 |
| Phospho-CREB (Ser133) | ab32096 | Rabbit | AB_731734 | | 1:5000 |
| β-hCG | ab9582 | Mouse | AB_296507 | | 1:1000 |
| E-cadherin | #3195 | Mouse | AB_2291471 | | 1:2000 |
| Cytokeratin 7 | ab181598 | Rabbit | AB_2783822 | | 1:8000 |
| β-actin | ab8227 | Rabbit | AB_2305186 | | 1:5000 |

**Table S2. Sequence of the siRNAs used in this study.**

| plasmid name | Primers | Sequence（5'~3'） |
| --- | --- | --- |
| si-GAB1-1 | Sense  Anti-sense | GACCGAUCUCCUGUGGAAA  UUUCCACAGGAGAUCGGUC |
| si-GAB1-2 | Sense  Anti-sense | GCAGAUGAGAGAGUGGAUUAU  AUAAUCCACUCUCUCAUCUGC |
| siNC | Sense  Anti-sense | UUCUCCGAACGUGUCACGU  ACGUGACACGUUCGGAGAA |

**Table S3. Sequence of the primers used for plasmids construction in this study.**

| plasmid name | Primers | Sequence（5'~3'） |
| --- | --- | --- |
| GAB1^WT^ | Forward primer  Reverse primer | CGGGATCC GGTTCCCGATCGAGTTCCTC  CGGAATTCCCACTCAGATCTCGTCTGCC |
| GAB1^lirMUT^ | Forward primer | GATCTGACTGGGGCTTGCGCCTCTTCCGCTTC-TGACAGAGGTTTTATGTC  GACATAAAACCTCTGTCAGAAGCGGAAGAGG-CGCAAGCCCCAGTCAGATC |
|  | Reverse primer |  |
